# Triglyceride-Glucose Index as a Predictor of Short-Term Outcomes in Acute Ischemic Stroke: A Prospective Observational Study From Bangladesh

**DOI:** 10.7759/cureus.109684

**Published:** 2026-05-26

**Authors:** Md. Emranul Huda, Muhammad Abdullah Al Amin, Ishrat Alam Zerin, Md. Atiqur Rahman, Md. Shamsuzzaman, Junaid Abdullah Jamiul Alam, S.M. Shihab Uddin, Mohammad Atiqur Rahman, Md Tauhidul Islam Chowdhury, Md. Badrul Alam Mondal

**Affiliations:** 1 Neurology, National Institute of Neurosciences and Hospital, Dhaka, BGD; 2 Endocrinology, National Institute of Neurosciences and Hospital, Dhaka, BGD

**Keywords:** acute ischemic stroke, insulin resistance (ir), modified rankin scale (mrs), mortality, short-term outcome, triglyceride glucose (tyg) index

## Abstract

Background

Acute ischemic stroke (AIS) is a primary cause of death and disability globally, imposing a significant burden in Bangladesh. Preliminary prognostic evaluation continues to be difficult, especially in resource-constrained environments. The triglyceride-glucose (TyG) index, an indicator of insulin resistance (IR), has surfaced as a possible predictor of poor outcomes in stroke. This study aimed to evaluate the association and predictive performance of the TyG index for short-term outcomes among patients with AIS. The primary objective was to assess its association and predictive ability for in-hospital mortality, while the secondary objective was to evaluate its association with 90-day functional outcome.

Methods

This prospective observational study was carried out in the Department of Neurology, National Institute of Neurosciences and Hospital (NINS), Dhaka, from January 2024 to June 2025. A total of 120 patients with radiologically confirmed first-ever AIS were included, of whom 118 completed the 90-day follow-up. Demographic data, clinical status, comorbidities, admission stroke severity, and laboratory values were gathered. Patients were observed for in-hospital mortality, and 90-day functional outcomes were evaluated using the modified Rankin Scale (mRS). The TyG index was calculated from fasting triglyceride and fasting plasma glucose levels measured within 24 hours of admission.

Results

The average age of participants was 63.38 ± 14.49 years, with 61.9% (n=73) being male. The in-hospital mortality rate was 28.8% (n=34). A higher TyG index was strongly linked with in-hospital mortality (median 9.49 vs. 8.85, p-value: 0.001). In multivariate logistic regression analysis, the TyG index remained an independent predictor of death (OR: 2.84; 95% CI: 1.43-5.64; p-value: 0.003). The ROC analysis showed moderate predictive performance (AUC: 0.680, p-value: 0.002), with an appropriate cutoff value of ≥8.87 (sensitivity 79.41%, specificity 51.19%). At 90 days, poor functional outcome was observed in 105 (89.0%) patients, which may reflect the tertiary-care hospital setting and inclusion of clinically more severe admitted stroke cases. A higher TyG index was significantly associated with poor functional outcome (OR: 2.21, 95% CI: 1.023-4.798; p=0.044). Patients with a higher TyG index had significantly lower survival rates, according to Kaplan-Meier analysis (log-rank p-value: <0.001).

Conclusion

A higher TyG index was significantly associated with increased mortality and poor functional outcomes among patients with AIS. However, its discriminative ability was moderate; therefore, the TyG index may be considered only as a simple, low-cost adjunct marker for early risk assessment rather than a standalone prognostic tool, particularly in resource-limited settings.

## Introduction

Globally, stroke remains a major public health problem and one of the leading causes of death and long-term disability [1,2]. Acute ischemic stroke (AIS) accounts for the largest proportion of new stroke cases and is associated with considerable mortality and functional impairment [3,4]. In Bangladesh, stroke represents a significant and growing health burden. According to World Health Organization statistics published in 2014, stroke was identified as the fifth leading cause of death in the country [5]. The Global Burden of Disease 2019 study identifies stroke as a major contributor to disability-adjusted life years (DALYs) and years of life lost in Bangladesh, with a disproportionately high burden compared to other South Asian countries [6,7]. Therefore, early prognostic assessment in AIS is clinically important, particularly in resource-limited settings, as it may help clinicians identify high-risk patients, guide timely management, prioritize available healthcare resources, and reduce the burden of mortality and long-term disability.

Insulin resistance (IR) has been recognized as a major risk factor for stroke, as it affects its occurrence, course, and prognosis through mechanisms such as the activation of platelets, the promotion of atherosclerosis, and the induction of hemodynamic disturbances [8]. The measurement of insulin and other indicators for IR is costly and not readily accessible in clinical practice, which significantly restricts their application. The triglyceride-glucose (TyG) index is a biological marker that is capable of predicting and screening a variety of diseases, particularly those that are associated with IR and metabolic syndrome. It is particularly useful for determining fasting triglycerides and glucose levels. The TyG index has been recognized as a reliable alternative indicator for IR [9]. The initial application was for cardiovascular diseases, and the connection with stroke remains somewhat restricted, primarily focusing on the incidence and recurrence of stroke, with a scarcity of research to support its association with prognosis [10]. Recent research has shown that AIS is consistently associated with worse short-term outcomes, including increased mortality, disability, and early neurological deterioration, due to elevated TyG levels. Throughout follow-ups of ≤3 and 12 months, meta-analyses of thousands of AIS patients have shown that elevated TyG is associated with poor functional outcomes and increased all-cause mortality [11-13].

Despite growing international evidence, data on the prognostic value of the TyG index among Bangladeshi patients with AIS remain limited. This evidence gap is important because differences in clinical severity, metabolic risk burden, delayed hospital presentation, and resource constraints may influence stroke outcomes in Bangladesh differently from high-resource settings. Although IR has an important role in stroke prognosis, its direct measurement is often impractical in routine clinical practice. Therefore, a low-cost marker derived from routinely available biochemical tests may have practical value as an adjunct tool for early risk assessment. Accordingly, this study was conducted to evaluate the association and predictive performance of the TyG index for short-term outcomes among patients with AIS admitted to a tertiary-care hospital in Bangladesh. The primary objective was to assess the association and predictive ability of the TyG index for in-hospital mortality. The secondary objective was to evaluate its association with 90-day functional outcome, measured by the modified Rankin Scale (mRS).

## Materials and methods

Study design, setting, and ethical considerations

This prospective observational study was conducted in the Department of Neurology at the National Institute of Neurosciences and Hospital (NINS), Dhaka, over a period of one and a half years from January 2024 to June 2025. Formal ethical approval for the study was obtained from the Institutional Review Board (IRB) of NINS (Reference: IRB/NINS/2023/320). Written informed consent was taken from all the participants or their relatives after explaining the aims and objectives of the study, and confidentiality of personal information was ensured throughout the research study in accordance with the Declaration of Helsinki.

Study population, inclusion, and exclusion criteria

Radiologically confirmed AIS patients admitted to the indoor section of the Department of Neurology were the study population. AIS was radiologically confirmed by neuroimaging (CT of the brain primarily and MRI of the brain if needed) within seven days of symptom onset. Among them, a total of 120 clinically diagnosed as first-ever ischemic stroke patients of both sexes and aged ≥18 years were included in the study following the purposive sampling technique. Patients with a history of acute myocardial infarction within the preceding three weeks, acute or chronic heart failure, malignancy, sepsis, multi-organ failure, or previous stroke with residual disability were excluded from the study.

Data collection and variables assessed

Following being recruited into the study, a detailed clinical history was taken, and comprehensive physical and neurological examinations were performed. A pretested semi-structured data collection sheet was used to collect the data from the study participants. Data collected included demographic variables (age, sex); risk factors such as smoking, alcohol consumption, hypertension, diabetes mellitus, coronary artery disease, and renal insufficiency; baseline clinical status (Glasgow Coma Scale (GCS)) [14]; time of stroke onset; in-admission stroke severity (assessed by National Institutes of Health Stroke Scale (NIHSS)) [15]; and TyG index. Relevant investigations, including neuroimaging (CT or MRI of the brain), electrocardiography, and biochemical analyses, were carried out for all patients. Venous blood samples were collected after an overnight fasting period of at least eight hours, and fasting plasma glucose (mg/dL) and fasting serum triglyceride levels (mg/dL) were measured within 24 hours of admission using an automated enzymatic method. All biochemical analyses were conducted using the Roche Cobas e801 automated analyzer (Roche Diagnostics International Ltd., Rotkreuz, Switzerland). The TyG index was calculated [16] as \[\mathrm{TyG\ index} = \ln\left(\frac{\mathrm{fasting\ triglyceride\ (mg/dL)} \times \mathrm{fasting\ plasma\ glucose\ (mg/dL)}}{2}\right)\]

Patient management, follow-up, and outcome assessment

**Figure 1:**
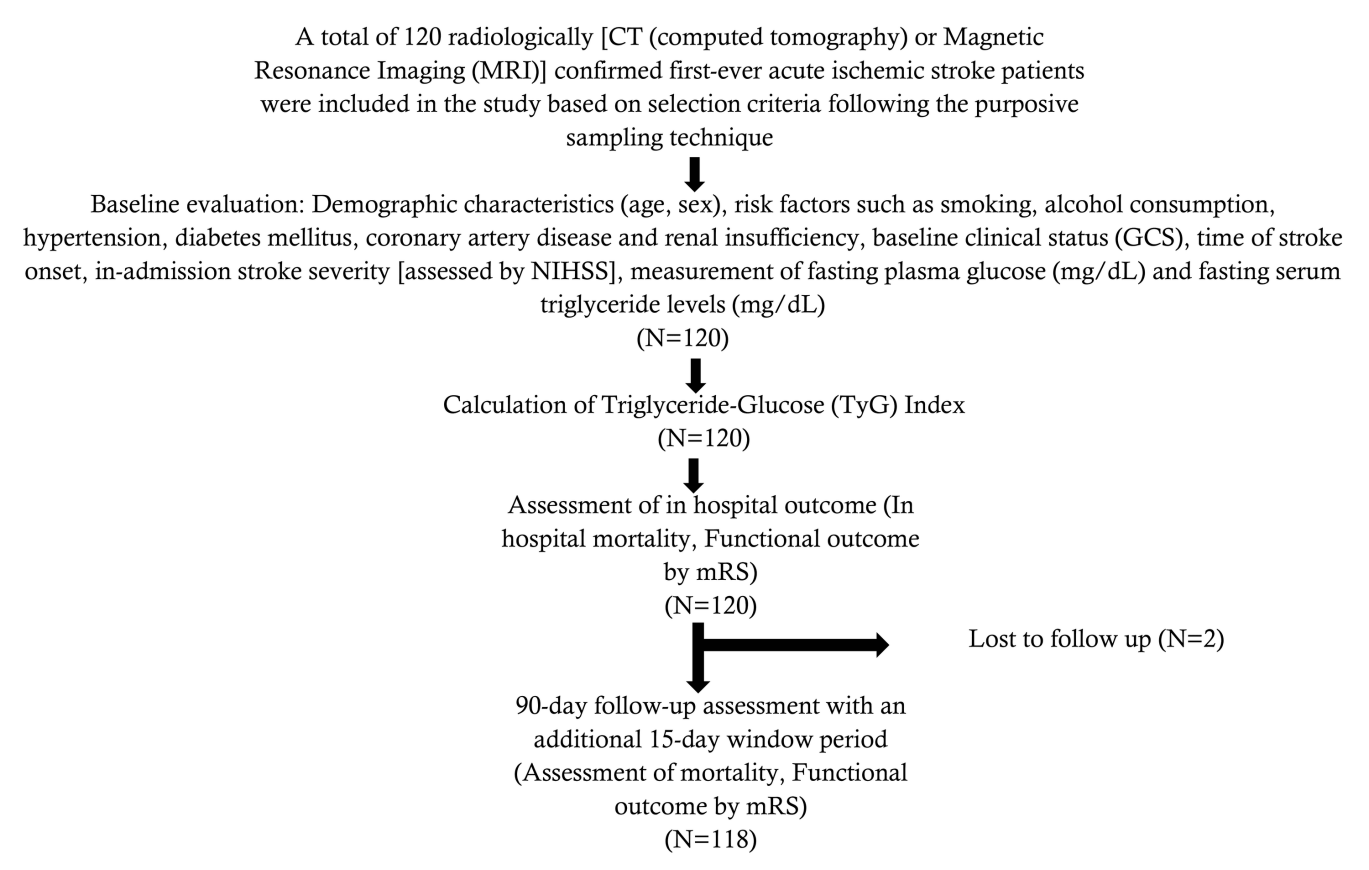
Study flow chart GCS: Glasgow Coma Scale; NIHSS: National Institutes of Health Stroke Scale; TyG Index: triglyceride-glucose index; mRS: modified Rankin Scale

All patients received standard care in accordance with established clinical guidelines. Discharge was undertaken upon clinical stabilization or improvement, either to home or to local healthcare facilities. Duration of hospital stay and in-hospital mortality were documented. Following discharge, patients were prospectively followed for 90 days to assess functional outcomes using the mRS [17], with an additional 15-day window period allowed for completion of the 90-day follow-up assessment. Functional outcomes were categorized as good (mRS 0-2) or poor (mRS 3-6). Follow-up evaluations were primarily conducted through face-to-face interviews at the stroke clinic; however, when this was not feasible, information was obtained from patients or their relatives either directly or via telephone. A total of 118 patients successfully completed the 90-day follow-up and were included in the final analysis. A study flow chart is shown in Figure 1.

Statistical analysis 

Data analysis was conducted utilizing the IBM SPSS Statistics for Windows, Version 23 (Released 2015; IBM Corp., Armonk, New York, United States). Continuous variables were reported as mean ± standard deviation (SD), median, and interquartile range (IQR) as indicated. Categorical variables were displayed as frequency and percentage. The Shapiro-Wilk test was utilized to evaluate the normality of continuous variables. For the purpose of comparative analysis, study participants were categorized based on their in-hospital outcomes (death versus alive) and their 90-day functional outcomes (good versus poor functional outcome). The chi-square test or Fisher’s exact test was employed to evaluate the relationships between categorical variables. An independent sample t-test was utilized to compare normally distributed continuous variables across groups, while the Mann-Whitney U test was employed for variables that did not follow a normal distribution. Logistic regression analysis was conducted to determine the factors linked to in-hospital mortality and poor functional outcomes at 90 days. Results were presented as OR accompanied by 95% CI. Nonetheless, multivariate analysis was not conducted for poor functional outcomes because of the limited number of significant predictors and to prevent model overfitting. An analysis of the receiver operating characteristic (ROC) curve was conducted to assess the predictive performance of the TyG index regarding in-hospital mortality. The area under the curve (AUC) with a 95% CI was computed, and the ideal cutoff value was established utilizing the Youden index. The calculations included sensitivity, specificity, positive predictive value (PPV), negative predictive value (NPV), and overall accuracy. The Kaplan-Meier method was utilized for survival analysis to evaluate 90-day survival rates among patients categorized by higher and lower TyG index (according to the established cutoff), with differences analyzed through the log-rank test. A p-value of less than 0.05 was deemed statistically significant.

## Results

The study population had a mean age of 63.38 ± 14.49 years, with the majority aged ≥50 years (85.6%, n=101) and a predominance of males (61.9%, n=73). A high prevalence of comorbid conditions was observed, including hypertension (71.2%, n=84), dyslipidemia (71.2%, n=84), diabetes mellitus (56.8%, n=67), and ischemic heart disease (26.3%, n=31). On admission, patients presented with severe clinical status, reflected by a low mean GCS score (8.14 ± 2.03) and a high mean NIHSS score (18.89 ± 6.54). The mean fasting glucose level was 186.62 ± 103.28 mg/dL, and the mean TyG index was 9.34 ± 1.14 (Table 1).

**Table 1:** Baseline characteristics of the study participants (N=118) TyG index: triglyceride-glucose index, GCS: Glasgow Coma Scale, NIHSS: National Institutes of Health Stroke Scale Data presented as mean ± standard deviation or frequency (percentage).

Baseline characteristics		Values
Age (years)	Mean ± SD	63.38 ± 14.49
	≥50	101 (85.6)
	<50	17 (14.4)
Gender	Male	73 (61.9)
	Female	45 (38.1)
Hypertension	Present	84 (71.2)
	Absent	34 (28.8)
Diabetes mellitus	Present	67 (56.8)
	Absent	51 (43.2)
Dyslipidemia	Present	84 (71.2)
	Absent	34 (28.8)
Ischemic heart disease (IHD)	Present	31 (26.3)
	Absent	87 (73.7)
GCS on admission	Mean ± SD	8.14 ± 2.03
NIHSS on admission	Mean ± SD	18.89 ± 6.54
Fasting glucose (mg/dL)	Mean ± SD	186.62 ± 103.28
Fasting triglyceride (mg/dL)	Mean ± SD	139.30 ± 82.18
TyG index	Mean ± SD	9.34 ± 1.14

**Figure 2:**
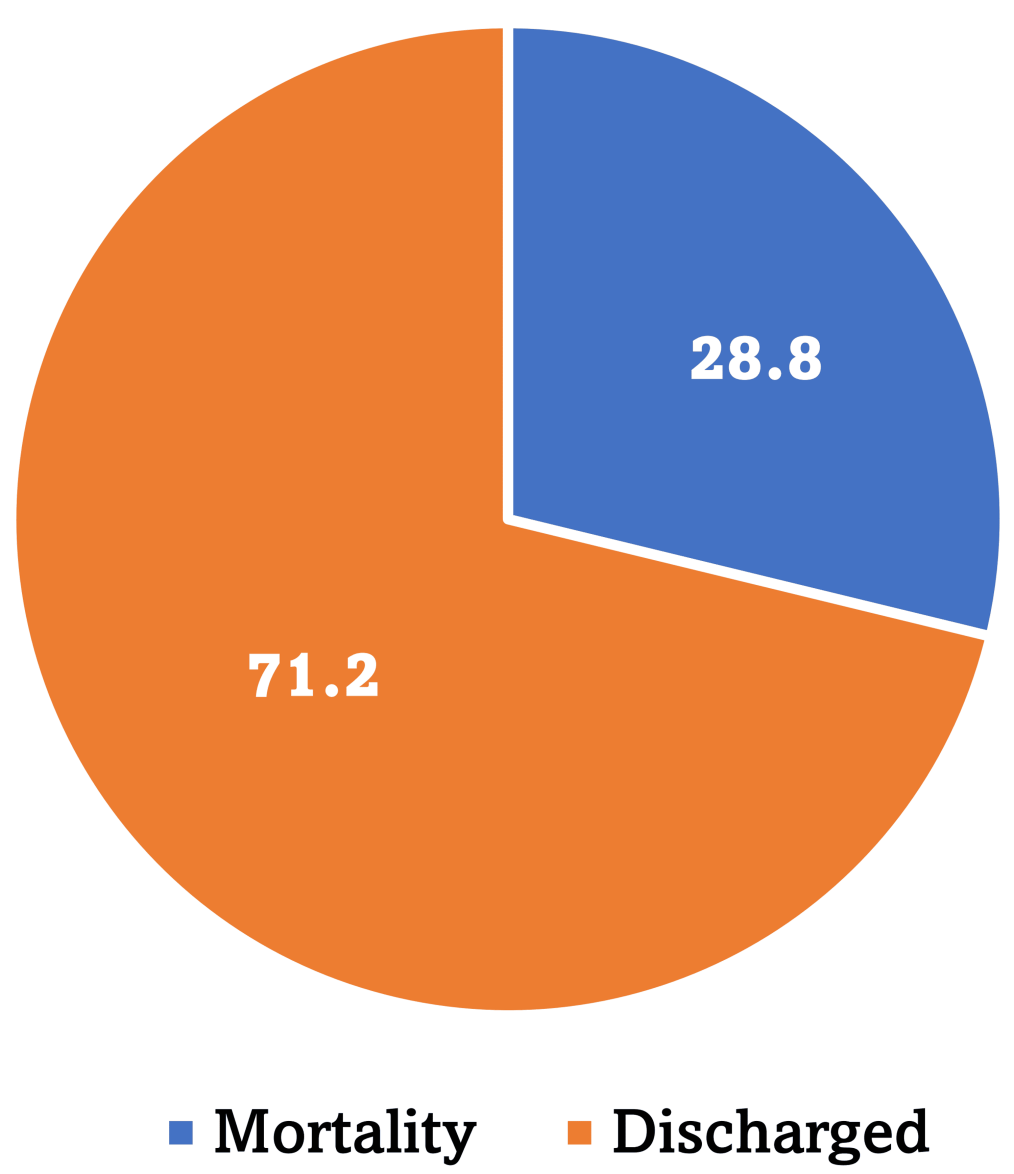
In-hospital outcome of the study participants (N=118)

Among the study participants, 34 (28.8%) patients died during hospital stay, while 84 (71.2%) were discharged (Figure 2).

There was no significant difference in age (64.32 ± 13.67 vs. 63.00 ± 14.87 years, p-value: 0.655) or sex distribution between non-survivors and survivors. Diabetes mellitus was significantly more prevalent among those who died (25 (73.5%) vs. 42 (50%), p-value: 0.019). On admission, non-survivors had significantly lower GCS scores (6.50 (5-8) vs. 8.50 (7-10), p-value: <0.001) and worse functional status as indicated by mRS (4.50 (3-5) vs. 5 (4-5), p-value: 0.002), while NIHSS showed a non-significantly higher value in the death group (23.50 (12.75-29.25) vs. 18 (14-19.75), p-value: 0.051). Among biochemical parameters, fasting glucose was significantly higher in non-survivors (203.85 (154.57-295.72) vs. 139.50 (107.05-199.80) mg/dL, p-value: <0.001), along with a higher TyG index (9.49 (8.89-11.68) vs. 8.85 (8.44-9.64), p-value: 0.001). Other variables did not show statistically significant differences (Table 2).

**Table 2:** Comparison of characteristics according to in-hospital outcome (N=118) TyG index: triglyceride-glucose index; GCS: Glasgow Coma Scale; NIHSS: National Institutes of Health Stroke Scale; HbA1c: glycated hemoglobin; SBP: systolic blood pressure; DBP: diastolic blood pressure; SD: standard deviation; mRS: modified Rankin Scale ^a^ Independent sample t-test; ^b^ Mann-Whitney U test; ^c^ chi-square test were performed. Data presented as frequency (%), mean ±SD, and median (interquartile range).

Variables		Death (n=34)	Alive (n=84)	p-value
Age (years)		64.32 ± 13.67	63 ± 14.87	^a ^0.655
Gender	Male	23 (67.6)	50 (59.5)	^c ^0.411
	Female	11 (32.4)	34 (40.5)	
Diabetes mellitus		25 (73.5)	42 (50)	^c ^0.019
Hypertension		28 (82.4)	56 (66.7)	^c ^0.088
Ischemic heart disease		9 (26.5)	22 (26.2)	^c ^0.975
Dyslipidemia		26 (76.5)	58 (69)	^c ^0.420
Current smoker		8 (23.5)	20 (23.8)	^c ^0.974
Medication history	Statin	15 (44.1)	23 (27.4)	^c ^0.078
	Antihypertensive drug	19 (55.9)	32 (38.1)	^c ^0.077
	Antidiabetic drug	20 (58.8)	42 (50)	^c ^0.385
Clinical findings on admission	SBP (mm of Hg)	155.88 ± 27.09	146.60 ± 24.49	^a^ 0.073
	DBP (mm of Hg)	90 (80-92.50)	90 (70-90)	^b^ 0.076
	GCS	6.50 (5-8)	8.50 (7-10)	^b^ <0.001
	NIHSS	23.50 (12.75-29.25)	18 (14-19.75)	^b^ 0.051
	mRS	4.50 (3-5)	5 (4-5)	^b^ 0.002
Biochemical parameters	HbA1c (%)	7.10 (5.99-9.72)	6.31 (5.70-7.37)	^b^ 0.088
	Fasting glucose (mg/dL)	203.85 (154.57-295.72)	139.50 (107.05-199.80)	^b^ <0.001
	Fasting triglyceride (mg/dL)	142.80 (82.67-221.80)	105.40 (78-165.60)	^b^ 0.097
	TyG index	9.49 (8.89-11.68)	8.85 (8.44-9.64)	^b^ 0.001

On univariate analysis, diabetes mellitus (OR 2.78, 95% CI 1.16-6.66, p-value: 0.022), admission NIHSS (OR 1.08, 95% CI 1.01-1.15, p-value: 0.017), and TyG index (OR 2.36, 95% CI 1.59-3.50, p-value: <0.001) were significantly associated with mortality. However, on multivariate analysis, only the TyG index remained an independent predictor of mortality (OR 2.84, 95% CI 1.43-5.64, p-value: 0.003), while diabetes mellitus and NIHSS lost statistical significance (Table 3).

**Table 3:** Logistic regression analysis for in-hospital mortality TyG index: triglyceride-glucose index, NIHSS: National Institutes of Health Stroke Scale Univariate and multivariate logistic regression were done. Data presented as odds ratio, 95% confidence interval.

Variables	Univariate analysis				Multivariate analysis			
	Odds ratio	95% confidence interval		p-value	Odds ratio	95% confidence interval		p-value
		Lower	Upper			Lower	Upper	
Diabetes mellitus	2.778	1.159	6.655	0.022	0.746	0.230	2.412	0.624
Admission NIHSS	1.081	1.014	1.152	0.017	1.063	0.988	1.143	0.100
TyG index	2.355	1.587	3.495	<0.001	2.842	1.432	5.643	0.003

**Figure 3:**
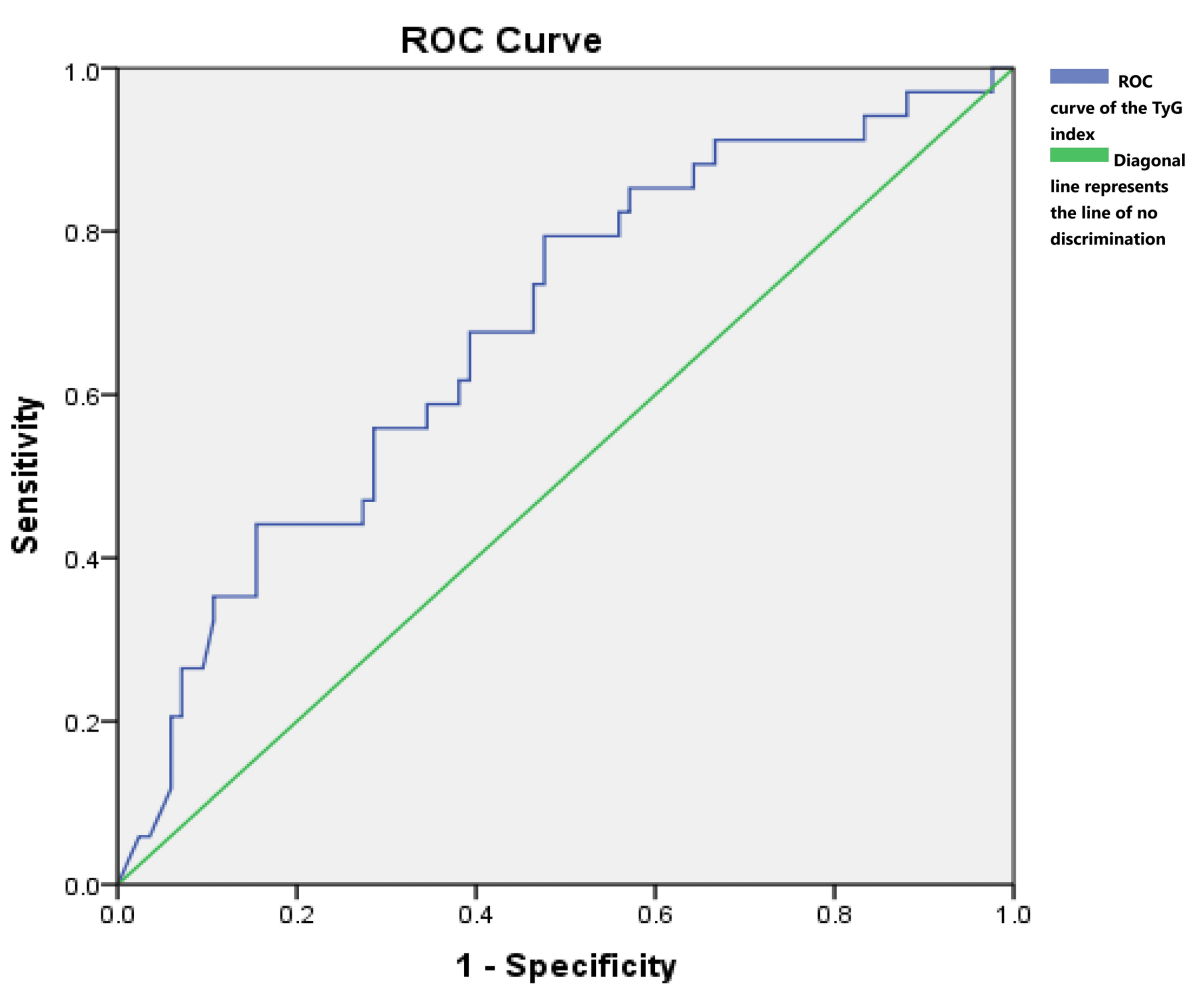
Receiver operator characteristics (ROC) curve for TyG index in predicting in-hospital mortality TyG index: triglyceride-glucose index

ROC curve analysis demonstrated that the TyG index had a moderate ability to predict in-hospital mortality, with an AUC of 0.680 (95% CI: 0.574-0.786, p-value: 0.002) (Figure 3).

A TyG index cutoff of ≥8.87, determined by the highest Youden index (Youden index: 0.318), demonstrated a sensitivity of 79.41% (95% CI: 62.10-91.30) and a specificity of 51.19% (95% CI: 40.04-62.26) for predicting in-hospital mortality. The PPV was 39.71% (95% CI: 33.28-46.51), while the NPV was relatively high at 86.00% (95% CI: 75.45-92.47), with an overall accuracy of 59.32% (95% CI: 49.89-68.27). These findings indicate moderate diagnostic performance, with better utility as a screening tool to rule out mortality rather than confirm it (Table 4).

**Table 4:** Diagnostic performance of the TyG index for predicting in-hospital mortality TyG index: triglyceride-glucose index Data presented as a percentage and 95% confidence interval.

Parameter	Value	95% confidence interval
Sensitivity	79.41	62.10 – 91.30
Specificity	51.19	40.04 – 62.26
Positive predictive value	39.71	33.28 – 46.51
Negative predictive value	86.00	75.45 – 92.47
Accuracy	59.32	49.89 – 68.27

There was no significant difference in age or sex distribution between good and poor functional outcome groups. Comorbidities, including diabetes mellitus, hypertension, ischemic heart disease, dyslipidemia, and smoking status, were also comparable between the groups. Among clinical parameters, systolic blood pressure was slightly higher in the poor outcome group (p-value: 0.027). Patients with good functional outcomes had significantly higher GCS scores (9 (8-11.50) vs. 8 (6.50-9.50), p-value: 0.001) and lower NIHSS scores (17 (14-17.50) vs. 19 (14-23.50), p-value: 0.008), indicating better neurological status at admission. Among biochemical parameters, the TyG index was significantly higher in the poor outcome group (9.05 (8.55-10.13) vs. 8.63 (8.40-8.99), p-value: 0.043), while HbA1c, fasting glucose, and triglyceride levels did not differ significantly (Table 5).

**Table 5:** Comparison of characteristics according to 90-day functional outcome TyG index: triglyceride-glucose index; GCS: Glasgow Coma Scale; NIHSS: National Institutes of Health Stroke Scale; HbA1c: glycated hemoglobin; SBP: systolic blood pressure; DBP: diastolic blood pressure; SD: standard deviation; mRS: modified Rankin Scale ^a ^Independent sample t-test, ^b ^Mann-Whitney U test, ^c^ chi-square test, and ^d ^Fisher’s exact test were performed. Data presented as frequency (%), mean ±SD, and median (interquartile range).

Variables		Functional outcome		p-value
		Good (n=13)	Poor (n=105)	
Age (years)		60.0±8.02	63.8±15.08	^a ^0.167
Gender	Male	7 (53.8)	66 (62.9)	^d ^0.556
	Female	6 (46.2)	39 (37.1)	
Comorbidities	Diabetes mellitus	6 (46.2)	61 (58.1)	^c ^0.412
	Hypertension	10 (76.9)	74 (70.5)	^d ^0.755
	Ischemic heart disease	2 (15.4)	29 (27.6)	^d ^0.510
	Dyslipidemia	8 (61.5)	76 (72.4)	^d ^0.517
	Current smoker	2 (15.4)	26 (24.8)	^d ^0.731
Clinical findings on admission	SBP (mmHg)	150 (130-175)	150 (135-160)	^b^ 0.027
	DBP (mmHg)	90 (75-100)	90 (80-90)	^b^ 0.576
	GCS	9 (8-11.50)	8 (6.50-9.50)	^b^ 0.001
	NIHSS	17 (14-17.50)	19 (14-23.50)	^b^ 0.008
Biochemical parameters	HbA1c	6.69 (5.85-8.49)	6.51 (5.70-8.51)	^b^ 0.806
	Fasting glucose (mg/dL)	115.70 (104.31-177.39)	163.80 (116.10-227.70)	^b^ 0.088
	Fasting triglyceride (mg/dL)	90 (70-122.95)	110.70 (78-189.10)	^b^ 0.084
	TyG index	8.63 (8.40-8.99)	9.05 (8.55-10.13)	^b^ 0.043

In the univariable logistic regression analysis, the TyG index was significantly associated with poor outcomes. Higher TyG values were associated with increased odds of poor outcome, with an OR of 2.215 (95% CI: 1.023-4.798, p-value: 0.044). In contrast, admission NIHSS showed a positive but statistically non-significant association with poor outcome (OR: 1.080, 95% CI: 0.979-1.192, p-value: 0.122). Admission systolic blood pressure was not associated with poor outcome (OR: 1.000, 95% CI: 0.978-1.023, p-value: 0.994) (Table 6).

**Table 6:** Univariate logistic regression for poor functional outcome at 90 days TyG index: triglyceride-glucose index, NIHSS: National Institutes of Health Stroke Scale; SBP: systolic blood pressure Univariate logistic regression was done. Data presented as odds ratio, 95% confidence interval

Variables	Odds ratio	95% confidence interval		p-value
		Lower	Upper	
Admission SBP	1.000	0.978	1.023	0.994
Admission NIHSS	1.080	0.979	1.192	0.122
TyG index	2.215	1.023	4.798	0.044

**Figure 4:**
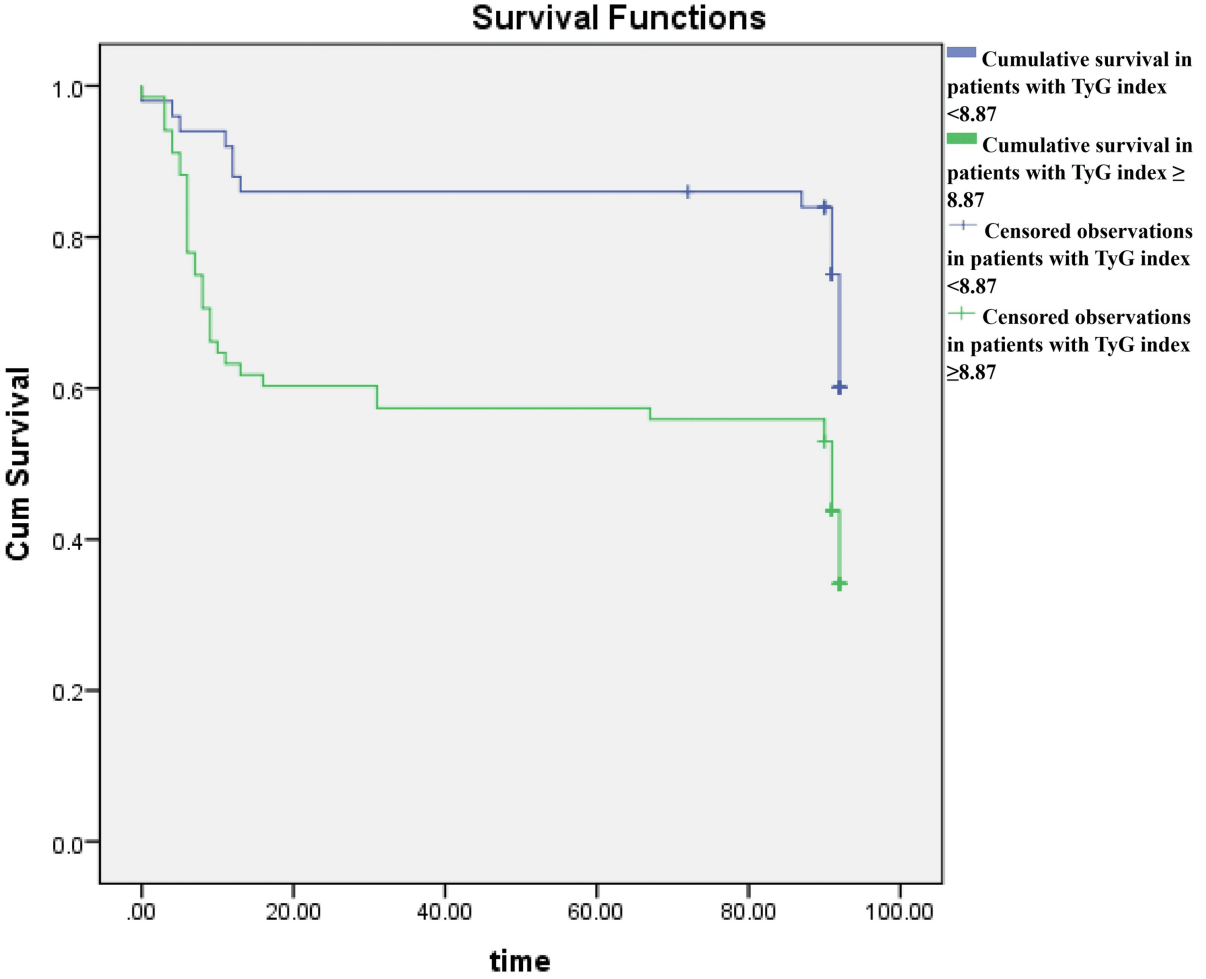
Kaplan-Meier analysis for 90-day mortality TyG index: triglyceride-glucose index

Kaplan-Meier analysis demonstrated that patients with a higher TyG index (≥8.87) had significantly reduced survival over 90 days compared to those with a lower TyG index (log-rank p < 0.001) (Figure 4).

## Discussion

This study evaluates the prognostic significance of the TyG index in foreseeable short-term outcomes, such as in-hospital mortality and 90-day functional status, in patients experiencing AIS. The baseline characteristics of our study population revealed that the majority of patients were elderly, with a notable predominance of males. A significant occurrence of comorbidities, especially hypertension, dyslipidemia, and diabetes mellitus, was noted. The results align with earlier research that has recognized older age and metabolic comorbidities as significant factors contributing to the burden of stroke [18,19]. The elevated mean NIHSS and diminished GCS at admission in our cohort indicate significant neurological impairment, which could account for the poor functional outcomes noted.

The in-hospital mortality rate in this study was 28.8% (n=34), which is higher than many international reports of first-ever AIS, although comparable to some hospital-based cohorts from low- and middle-income settings. Cai et al. (2023) reported an in-hospital mortality rate of 19.0% among ischemic stroke patients, particularly in those with elevated metabolic risk profiles [20]. The higher mortality observed in our cohort may partly reflect late hospital presentation, limited access to advanced stroke treatment, and a high burden of comorbid conditions. In addition, referral and selection bias should be considered, as this study was conducted in a tertiary-care neuroscience hospital where more clinically severe patients are likely to be admitted or referred. The high mean NIHSS score and low mean GCS score at admission further support the likelihood that the cohort included a greater proportion of severe stroke cases.

In our comparison of survivors and non-survivors, we identified significant associations with mortality, including diabetes mellitus, lower GCS, higher NIHSS, elevated fasting glucose, and a higher TyG index. The results are consistent with earlier studies indicating that neurological severity and metabolic dysregulation are key factors influencing stroke outcomes [21,22]. The elevated TyG index in non-survivors indicates that IR could play a role in worse outcomes via mechanisms including endothelial dysfunction, inflammation, and prothrombotic states.

In the regression analysis, the TyG index emerged as a strong predictor of mortality. Although diabetes mellitus and NIHSS were significant in univariable analysis, they lost significance in multivariable analysis, while the TyG index remained independently associated with in-hospital mortality. This finding is consistent with previous studies, such as Toh et al. (2022) and Cai et al. (2023), which reported that a higher TyG index independently predicted mortality in AIS patients [20,23]. The loss of significance of NIHSS and diabetes mellitus after adjustment may be due to the overlapping pathophysiological pathways captured by the TyG index, as it reflects both glycemic and lipid abnormalities linked to IR.

The ROC curve analysis in our study demonstrated that the TyG index had a moderate predictive ability for in-hospital mortality, with an AUC of 0.680. This is comparable to findings from other studies, where the predictive performance of TyG ranged from modest to moderate [24,25]. The identified cutoff value of ≥8.87 showed high sensitivity (79.41%) but moderate specificity (51.19%), indicating that the TyG index may be more useful as a simple adjunct marker to complement clinical assessment rather than as a standalone prognostic tool for decision-making. The relatively high NPV further supports its utility in ruling out mortality risk.

The functional outcome study indicated that a significant percentage of patients (89%) experienced poor outcomes at 90 days, reflecting a considerable impairment burden. This study was conducted in a tertiary-care neuroscience hospital, where clinically severe AIS cases are more likely to be admitted or referred. Patients with unfavorable outcomes had lower GCS scores, higher NIHSS scores, and higher TyG index values at admission, indicating greater neurological severity and metabolic risk in this subgroup. The results align with the research conducted by Lin et al. (2022) and Lee et al. (2021), which indicated that an elevated TyG index correlates with poorer functional outcomes post-stroke [26,27]. The correlation between the TyG index and adverse functional outcomes may be attributed to IR's role in facilitating neuroinflammation, oxidative stress, and compromised neuronal recovery.

However, in the univariable logistic regression for poor functional outcome, only the TyG index showed a statistically significant association, while NIHSS and SBP were not significant predictors after adjustment. This may be partly due to the limited number of patients with good functional outcomes in our study, which could reduce statistical power. Additionally, NIHSS and GCS are closely related measures of stroke severity, and potential multicollinearity may have influenced the regression results.

The Kaplan-Meier survival analysis further supported the prognostic value of the TyG index, showing significantly reduced survival among patients with higher TyG values. This finding is in agreement with previous studies by demonstrating that an elevated TyG index is associated with increased mortality and adverse outcomes over time [28,29]. The consistent association across different analytical approaches strengthens the validity of our findings, and these results provide strong evidence supporting their biological validity. IR exacerbates ischemic brain injury and impedes recovery by promoting atherosclerosis, increasing inflammatory cytokine production, enhancing platelet activation, and contributing to endothelial dysfunction [30]. The TyG index, which functions as a surrogate marker of IR, incorporates these metabolic disturbances and offers a practical risk stratification approach. In general, our results are in agreement with the international literature, despite the existence of some discrepancies in the strength of the association and predictive performance. These discrepancies may be attributable to variations in the severity of strokes, healthcare settings, study populations, and treatment availability.

Limitations

This study has several limitations. It was conducted in a single tertiary-care neuroscience hospital with a relatively small sample size, which may limit generalizability. The hospital-based design and purposive sampling may have introduced referral or selection bias, as more clinically severe stroke cases are likely to be admitted or referred to such a specialized center. The high mean NIHSS score and low mean GCS score at admission support this possibility. Therefore, the high in-hospital mortality rate and high proportion of poor functional outcomes may partly reflect the severity profile of the enrolled patients rather than the prognosis of first-ever AIS in the general population. Although the TyG index remained independently associated with in-hospital mortality, the multivariable model was limited by sample size and number of events. For the 90-day functional outcome, multivariable analysis was not performed because of the small number of patients with good outcomes, which limited inferential strength and increased the risk of overfitting. Important factors such as time to hospital presentation, acute stroke management, reperfusion therapy, rehabilitation, and post-discharge care were not fully assessed. IR was not directly measured, and the TyG index was not compared with established indices such as homeostasis model assessment for IR (HOMA-IR) or the hyperinsulinemic-euglycemic clamp. Finally, some follow-up data were obtained by telephone, which may have introduced information bias.

## Conclusions

This study demonstrated that an elevated TyG index was significantly associated with higher in-hospital mortality and poorer short-term functional outcomes among patients with AIS. After adjustment for selected clinical covariates, the TyG index remained independently associated with in-hospital mortality, suggesting that it may provide additional prognostic information in this patient group. However, given its moderate discriminative accuracy, limited model adjustment, and the possibility of referral or selection bias related to the tertiary-care hospital setting, its clinical interpretation should remain cautious. The TyG index may therefore be regarded as a simple, inexpensive, and readily available adjunct marker for early risk assessment, but not as a standalone prognostic tool. Larger multicenter studies with broader patient populations and more comprehensive adjustments for clinical confounders are required to validate these findings and define their role in routine stroke care.
